# On the Destruction and Humanitarianisation of the Health System in Gaza and the Need for a Biopolitical Bioethics

**DOI:** 10.1111/bioe.70118

**Published:** 2026-06-22

**Authors:** Mohammad Salaymeh, Arianne Shahvisi, Sohail Jannesari

**Affiliations:** ^1^ Médecins Sans Frontières Palestine; ^2^ Brighton and Sussex Medical School United Kingdom; ^3^ Imperial College London United Kingdom

**Keywords:** Gaza, health systems, Israel, necropolitics, Palestine

## Abstract

This paper examines Israel's destruction and ‘humanitarianisation’ of Palestinian health systems, arguing that this should be understood as an instance of ‘necropolitics,’ as conceived by Achille Mbembe. We review the extensive, long‐term destruction of health systems in Palestine before 7 October 2023 and the catastrophic acceleration of that destruction in Gaza in the years since. We show that this process is central to Israel's project of limiting Palestinian life to what Mbembe calls a ‘death world’, a space where power is exercised through the systematic exposure of a population to death, and that it serves the security and economic agendas of Israel and its allies. By reducing Palestinians to death‐like living conditions through the destruction and subsequent humanitarianisation of health infrastructure, Israel optimises its control over the remaining population of Gaza. We argue that necropolitics, and biopolitics more generally, are vital yet underused tools in bioethics, without which situations of extreme oppression cannot be adequately theorised.

## 1 Introduction

Access to health systems is a core determinant of a person's health and wellbeing, and control of health systems is key to national sovereignty. Those seeking to control, oppress, or eliminate ‘threatening’ or ‘undesirable’ populations can achieve this end by attacking health infrastructure and restricting access to healthcare services. Destroying the health of a population diverts the energies of an oppressed people towards surviving, which reduces their capacity for resistance and makes them more compliant to, and dependent on, the oppressing power [1]. It has the advantage of being less morally troubling than directly committing genocide through mass killing and often produces a desperate, exploitable workforce.

Scholars of biopolitics recognise the important link between the (ill‐)health of the body and the (ill‐)health of the population, and the unique opportunities this presents for discipline and control [2, 3, 4, 5, 6]. This recognition grants their analyses greater precision and nuance in understanding the operation of power and its effects on health. This work adds to the growing literature that consciously opens up bioethics to biopolitical considerations, stepping away from the more traditional remit of the discipline that is, the ethics of individual clinical encounters and new biomedical technologies. In this work, we reject the division between (bio)ethics and (bio)politics, and one of our aims is to argue for the necessary continuity of the two. In doing so, we endorse the position taken by Lederman and Lederman and also Shahvisi, who argue that bioethicists have responsibilities to engage with political issues which have bioethical causes or consequences, and follow the still lightly‐trodden path of biopolitical bioethics typified by the work of Rose, Petryna, Fassin, and Ticktin, Mills, and Bishop (among others) [4, 7, 8, 9, 10, 11, 12, 13, 14, 15].

In this article, we examine the current and historical destruction and ‘humanitarianisation’ of Palestinian health systems by the state of Israel through a biopolitical lens. Specifically, we apply the theory of ‘necropolitics’ developed by philosopher Achille Mbembe to understand the importance of health destruction to Israel's project of limiting Palestinian life to a ‘death world’ [3]. We show that alongside the genocide in the Gaza Strip that followed the Hamas‐led attacks of 7 October 2023, Israel has been conducting a deliberate campaign of health destruction and humanitarianisation that will have long‐term necropolitical effects, thereby optimising their control of the remaining population of Gaza in order to serve the security and economic agendas of themselves and their allies. We show that this biopolitical analysis is a necessary part of any morally and epistemically defensible bioethics.

The article is structured as follows. Section 2 provides the context for the paper's analysis, describing the state of Palestinian health systems before and after 7 October 2023 and demonstrating that they have been successively ‘humanitarianised.’ Section 3 introduces biopolitics more generally, and necropolitics specifically, and then applies necropolitics to the situation described in section 1, arguing that even in the context of genocide, there is a broader necropolitical agenda. Section 4 argues that necropolitics and biopolitics more generally are an important and underused part of the bioethics toolkit without which situations of extreme oppression cannot be properly theorised. Section 5 offers some practical suggestions for resisting and analysing the effects of humanitarianzation and necropolitics.

While the paper is limited to Palestine, and the Gaza Strip more specifically, parts of its analysis may extend to other settings in which healthcare infrastructure has been weaponized, such as in Sudan [16], Syria [1], and Ukraine [17].

### 1.1 A Reflexive Note

While this is a work of theoretical bioethics, and therefore proceeds via argument rather than empirical research, the tone and content of that argument are determined by the authors' identities and experiences. We therefore follow the practice urged by Ives and Dunn of ‘acknowledging openly that bioethics is an embedded socio‐cultural practice’ and offering additional information about the authors' backgrounds and motivations with the objective of being transparent about our personal interests, so that any bias can be more easily accounted for, but also reminding readers that academic work is very often entwined with the real, material lives of its writers [18]. Therefore:

Mohammad Salaymeh is a Palestinian from Jerusalem who works for Médecins Sans Frontières as a humanitarian affairs officer focusing on Gaza. Their work focuses on understanding how Israeli settler colonialism develops in historical Palestine and exploring the best methods for Palestinians to counter colonialism in order to continue existing in their land.

Sohail Jannesari is a British‐Iranian migrants' rights lecturer interested in opening up more academic spaces for Palestinian perspectives. Their involvement in this paper is personal, political and academic. They are conscious that their academic career is benefitting from being a co‐author with Mohammad, and find that there are tensions between their differing motivations. Key to navigating them is giving this philosophical paper a detailed historical grounding and providing concrete examples of solidarity.

Arianne Shahvisi is a Kurdish‐British philosopher whose work focusses on social justice, with particular attention to gender, race, and migration. Kurds are a persecuted group subject to state violence and genocide [19]. Shahvisi sees connections between these struggles and feels a sense of responsibility to both sets of people. They have been involved in activism in solidarity with Palestinians, and have worked with a youth organisation in Palestine.

## 2 The Humanitarianisation of Health Systems in Gaza

In this section, we tell the story of how the health system of Gaza became ‘humanitarianised’. We begin by offering some background on humanitarianism and humanitarianisation, and then describe the state of Palestinian health systems before and since 7 October 2023.

### 2.1 Humanitarianisation

The modern humanitarian system dates back to the 19th century. After witnessing the mass casualties in the Battle of Solferino in 1859, Swiss philanthropist Henry Dunant called for a neutral humanitarian actor to provide impartial healthcare to all warring parties [20]. Dunant's efforts led to the creation of the first humanitarian organisation, The International Committee of the Red Cross (ICRC) in 1863 and the drafting of the first Geneva Convention in 1864, which was a precursor to modern international humanitarian law [21].

The principle of neutrality was foundational to the humanitarian enterprise, and aid organizations were positioned as impartial actors providing assistance based solely on need. This commitment to neutrality was intended to secure access to vulnerable populations across conflict lines while maintaining the safety of humanitarian workers. Humanitarian organizations embraced this position, functioning as apolitical entities that deliberately avoided taking sides in hostilities or engaging in controversies of a political, racial, religious, or ideological nature. However, this strict adherence to neutrality faced significant challenges as humanitarian crises exposed the limitations of silence in the face of atrocities. In 1971, a group of French doctors left the ICRC during the Nigerian Civil War (Biafra conflict) in protest at the organisation's attempted neutrality and lack of public advocacy, a move which led to the establishment of *Médecins Sans Frontières* (MSF) [22]. MSF added a new dimension to humanitarian action: the moral responsibility of bearing witness, or *témoignage*. Bearing witness includes the documentation and publicising of humanitarian catastrophes, adding a layer of advocacy to humanitarian action [23].

More recently, critics have argued that humanitarian actors help sustain a neocolonial order, wielding soft power under the guise of neutral aid. This, they contend, creates openings for Western states to influence countries in Africa, Asia, and Latin America where more overt or direct intervention would face strong resistance [24]. The creation of the United Nations Relief and Works Agency for Palestine Refugees in the Near East (UNRWA) in 1949 drew criticism from Palestinians who saw it as the ‘humanitarianisation’ of the question of Palestine. Critics urged that international efforts focus instead on land‐oriented restorative justice, rather than reducing Palestinians to a population dependent on overseas benefactors for ‘a sack of flour and a can of cooking oil [25].’ The arrival of international NGOs in the occupied Palestinian territories (oPt) during the First Intifada led to further critique, with Palestinians accusing humanitarian groups and NGOs of depoliticizing and decontextualizing the cause of Palestinian civil society [26]. Others have argued that these organizations perpetuate the Israeli occupation by creating a humanitarian biosphere that facilitates Israeli state violence under the camouflage of humanitarianism [27].

‘Humanitarianisation’ therefore refers to the process by which long‐term social and political problems are reframed as humanitarian emergencies requiring immediate technical solutions rather than resolution of the root social, political, and economic determinants. In the Palestinian context, and particularly in Gaza, this is seen in the transformation of what is fundamentally a political crisis into a series of humanitarian interventions focused on emergency healthcare provision. The Palestinian healthcare system has become increasingly dependent on international humanitarian organizations, donor funding cycles, and emergency medical relief. This shift has restructured healthcare from a sustainable public service into a patchwork of humanitarian projects, where medical infrastructure exists in a perpetual state of crisis management rather than development, and where healthcare workers are forced to operate within the constraints of humanitarian frameworks rather than building robust health systems designed for longevity and self‐sufficiency. In what follows, we give a brief history of humanitarianisation in Gaza.

### 2.2 The State of Palestinian Health Systems Before 7 October 2023

The tradition of the oppressed teaches us that the ‘state of emergency’ in which we live is not the exception but the rule. —Walter Benjamin [28]

The *Nakba*, (‘catastrophe’ in Arabic) refers to the ethnic cleansing and forced displacement of around 750,0000 Palestinians from their ancestral lands in historical Palestine during the establishment of the State of Israel in 1948 [29]. In 1967, after Israel's surprise victory in the 6‐Day war, the Israeli state occupied what was left of historical Palestine. The occupied Palestinian territories (oPt)—the West Bank, the Gaza Strip, and East Jerusalem and the two million Palestinians within those areas—came under direct military Israeli control. The Israeli defense minister at the time, Moshe Dayan, declared that Israel's central problem in the oPt was the question of how to control millions of Palestinians living under its rule [30].

Shlomo Gazit, one of Dayan's lieutenants, was appointed to oversee the political, security, and economic affairs of the newly oPt and find a framework for control. Gazit designed the main underpinnings of the Israeli occupation, framing it as an ‘enlightened’ occupation [31] to create a civilizational separation between the ‘backward’ Palestinians and the ‘enlightened’ ‘civilised’ Israelis [32], who must improve (and control) the lives of Palestinians through benevolent humanitarian efforts. ‘Humanitarianising’ the Question of Palestine meant recasting the expulsion of Palestinians from historical Palestine as a decontextualized humanitarian issue, rather than situating it within Israel's project of settler colonialism and nation‐building [33]. This reframing allowed Israel to redefine the Palestinian plight, presenting the refugees of the 1948 Nakba in humanitarian terms and portraying those in refugee camps as a ‘displaced population’ in need of resettlement outside Palestine [34, 35]. The logic of humanitarian displacement has persisted and evolved, as seen in US President Donald Trump's proposal to relocate Palestinians from Gaza to Egypt, Jordan, or other locations beyond Palestine [36].

Between 1967 and 1993, Israel consolidated its occupation regime, exemplified by the establishment of the Unit for the Coordination of Governmental Operations in the Territories. (COGAT), with Shlomo Gazit as one of its first leaders [32]. Israel created and refined an extensive bureaucracy and policies to control and subjugate millions of Palestinians, reducing their quality of life to a bare minimum by restricting access to healthcare, education, employment and freedom of movement [37].

COGAT control of the Palestinian healthcare system was characterised by deliberate under‐development, engineering Palestinian reliance on Israeli hospitals for advanced care by ensuring inadequate budgeting for Palestinian healthcare centres, restrictions on licenses for new health and medical projects, and frequent referrals to Israeli hospitals for specialist care [38]. These referrals have allowed the Israel system to pass as a compassionate ‘humanitarian’ actor. Israeli control was further consolidated through the establishment of the permit system, which punished Palestinians with histories of political activity by blacklisting them and their family members, preventing the issuing of medical permits, and thereby restricting access to healthcare [39, 40]. In 2022 a 19‐month‐old girl in Gaza, Fatima al‐Masri, died after waiting 5 months for Israel to issue a permit so she could travel for life‐saving heart surgery. Her father recalled that ‘They kept saying the application was “under review, under review” and then she died’ [41].

Many Palestinians resisted this control through the formation of Palestinian health committees in the mid 1980s, who worked to ‘supplement the decayed and inadequate health infrastructure caused by years of Israeli military occupation [42]’ with the aim of reclaiming Palestinian health sovereignty. The work of many Palestinian Popular Committees took inspiration from other Global South anti‐colonial movements, and focused on providing primary and public health to Palestinians, emphasizing holistic care that addressed social, political, and economic determinants [43]. These committees continued to grow, and turned into full organisations like the Union of Palestinian Medical Relief Committees (today's Palestinian Medical Relief Society) [42], and the Union of Health Work Committees, who now run Al‐Awda hospital in Gaza [44].

In December 1987, the Israeli military occupation led to the first Palestinian uprising, the ‘First Intifada’, a prolonged wave of protests, civil disobedience, and collective resistance led by Palestinians inside the oPt and Israel [45]. In response, the Israeli authorities implemented the policy of ‘force, might, and beatings,’ which included what became known as the ‘break‐their‐bones’ strategy—a systematic use of beatings against Palestinian protesters [46]. Israeli occupation forces also relied heavily on collective punishment, most notoriously the imposing of full curfews on Palestinian towns and villages [47]. One such curfew, imposed on the Palestinian town of Beit Sahour [48] lasted 42 days. The First Intifada was defined by the ubiquitous Israeli checkpoints, which proliferated around the oPt, erasing the limited freedom of movement of Palestinians, tightening the Israeli occupation's grip on Palestinian life, and formalising the Israeli permit system [49]. The First Intifada concluded in 1993 with the signing of the Oslo Peace Accords, marking the establishment of the Palestinian Authority (PA) which created several Palestinian bureaucratic structures, including the Palestinian Ministry of Health (MoH) in both Gaza and the West Bank. Even after the formal establishment of the Palestinian Authority, Israel permanently maintained the checkpoint system developed during the First Intifada, despite the onset of a peace process [50, 51].

The First Intifada received widespread media coverage, attracting the attention of international NGOs in the oPt, which led to the PA's MoH and international NGOs taking charge of much of the bureaucracy and services previously provided by COGAT and the Palestinian healthcare committees [52]. The Palestinian Ministry of Health (MoH) inherited a fragmented healthcare system and became increasingly reliant on international aid. This reliance made the system vulnerable to restrictive reforms, including the elimination of health committees [26]. Despite the MoH's efforts to improve healthcare capacity by increasing the number of Palestinian healthcare workers and establishing several medical schools in Gaza and the West Bank, the PA was never able to reform the Palestinian healthcare system or liberate it from Israeli control and reliance [52]. Subsequent political events produced further challenges. Hamas won the Palestinian popular vote in the 2006 PA elections in Gaza and the West Bank, but was unable to form a joint government with Fatah, a more established Palestinian faction that played a central role in the Oslo Peace process and led the PA since its establishment [53]. In 2007, the political deadlock between the two Palestinian parties led to armed clashes, concluding with Hamas ousting Fatah from the Gaza Strip and taking full control of it, and Fatah retaining control of the West Bank [54]. This fracture between the Palestinian parties led to the establishment of two separate PAs: The Fatah‐led PA in the West Bank, with its own MoH, and the Hamas‐led PA in the Gaza Strip, with its own separate MoH, further fragmenting the healthcare system [55]. In response to Hamas's takeover of the Gaza Strip, Israel imposed a full blockade in 2007, intensifying the partial restrictions that had been in place since 1991 [56].

While it is important to understand the shared architecture of occupation across the oPt as a unified political project, Gaza has always had a distinct status within Israeli policy [57]. From 1967 onward, the dense concentration of refugees in Gaza who were displaced by the 1948 Nakba were framed as a demographic and security threat, prompting deliberate efforts to depopulate the Strip by degrading living conditions [35]. Israeli officials, including Moshe Dayan [58] and later Ariel Sharon [59], viewed Gaza's refugee camps as potential and actual sites of resistance to be dismantled through both structural neglect and direct military force. Sharon's brutal campaigns in the 1970s, including bulldozing the homes of already‐displaced Palestinians in refugee camps in Gaza, exemplify this logic of erasure [60]. After Hamas's electoral victory in 2006 and subsequent takeover in 2007, this policy of containment intensified, with the blockade functioning as a mechanism to render Gaza unliveable while maintaining Israel's control over borders, movement, and essential infrastructure [61].

The Gazan healthcare system deteriorated further due to continuous eruptive attacks on the Strip, alongside an increasingly restrictive blockade [62]. Since the imposition of the blockade in 2007, many hospitals and healthcare clinics in the Gaza Strip have been repeatedly targeted by Israeli airstrikes during successive military operations [63]. Beginning with Operation Cast Lead in 2008 and 2009, these strikes were often justified by Israeli authorities on the grounds that Hamas was allegedly using medical facilities as ‘human shields’ [64]. Claims about the use of ‘human shields’ or ‘hospital shields’ to justify the destruction of healthcare facilities have never been substantiated and are widely critiqued as a legal strategy to justify attacks in densely populated areas [65]. As Gordon and Perugini note, such framing erodes protections for medical infrastructure and normalises its targeting under the guise of military necessity [65]. As a result, Palestinian hospitals, despite having many locally trained doctors and healthcare workers, could not provide sufficient essential care to Gazans [66]. Patients with complex health needs began to rely on Israel to issue medical permits in order to leave Gaza and access healthcare in the West Bank, East Jerusalem, Israel or abroad [67]. Since 2007, supplies of essential drugs and equipment have been critically low and many international NGOs have devoted significant effort to negotiating the entry of crucial medical supplies into Gaza [66]. The cycle of smaller recurring wars and a restrictive blockade on Gaza's healthcare created a permanent humanitarian crisis inside the Gaza Strip, opening the door for humanitarian NGOs to settle inside the Strip, propping up Palestinian hospitals and sometimes replacing them [68].

Most international NGOs have historically refrained from challenging Israel's occupation policies, aiming instead (at best) to mitigate their effects [69]. Many have actively reinforced Israel's permit regime by focusing on securing medical permits for Palestinians in Gaza and the West Bank [70], compiling files and medical reports that frame Palestinians primarily as victims [71, 72]. This approach has enabled the Israeli Coordination of Government Activities in the Territories (COGAT) to present the limited permits it issues as symbols of humanitarian benevolence, while rejecting the majority on purported security grounds [73].

In practice, numerous international humanitarian organisations operating in the occupied Palestinian territory (oPt) have assumed administrative roles within the architecture of occupation [74]. By avoiding direct engagement with the core issue—Israel's occupation and blockade of Gaza—many aid groups have inadvertently contributed to sustaining and co‐developing the blockade, particularly through mechanisms of healthcare control [75].

### 2.3 The State of Palestinian Health Systems Since 7 October 2023

On 7 October 2023, Hamas and other Palestinian factions broke through Gaza's borders and carried out a massacre in Israeli kibbutzim, nearby towns, and at the Supernova Music Festival near the Gaza Strip border, killing nearly 1200 Israelis and taking around 250 people as hostages [76]. In response, Israel has dramatically escalated its violence within the Gaza Strip, producing a situation described by the UN Special Rapporteurs as ‘consistent with genocide [77, 78]’. The Israel Defense Forces (IDF) have systematically targeted Palestinian hospitals, again under the pretext that Hamas uses the facilities as ‘hospital shields’ [65]. At the time of writing, only 18 out of 36 Palestinian hospitals in Gaza remain partially operational [79]. The attacks have also decimated Gaza's medical workforce and ambulance services. ‘Our mission is humanitarian, we provide humanitarian service only, and we were targeted without any reason, without any excuse,’ said paramedic Talal Taha, after an Israeli air strike on emergency vehicles killed three of his colleagues during a rescue operation.^1^

In October 2023, Israel issued an evacuation order for 1.1 million Palestinians in and around Gaza City, signalling its intention to conduct a mass displacement operation [80]. The plan was thwarted by Egypt's refusal to open its borders [81]. Hospitals in Gaza were also ordered to evacuate, despite the impossibility of relocating critically ill patients. Medical teams categorically refused. ‘Everyone is determined that they will not evacuate’, said Dr. Khalil al‐Degran of al‐Aqsa Martyrs Hospital. ‘How can we transfer patients in intensive care or those undergoing surgery? If the Israelis want to bomb the hospitals then it will be over our heads as we stand by our patients [82].’ Israel's approach shifted later that month, once it became evident that the 2.1 million Palestinians in Gaza would not leave the strip en masse^2^. The revised strategy appears to be to leverage humanitarian aid organisations and international humanitarian law (IHL) to create a more restrictive environment for Palestinians, tightening Israeli control over the population while influencing the terms of humanitarian assistance [27]. In Rafah, Israel has reportedly discussed plans to construct detention centers to manage and confine displaced Palestinians under military supervision. The proposal has been described by former Israeli prime minister Ehud Olmert as a "concentration camp" [83].

Israel's instrumentalization of humanitarian aid to reinforce its occupation regime was aided by the establishment of the so‐called ‘Humanitarian Zone.’ The Zone was declared on 29 October 2023, by COGAT and the IDF, who presented it as a measure to ‘facilitate humanitarian efforts’ in Gaza [84]. The Humanitarian Zone functions as a zone in which human life is severely restricted and entirely dependent on outside forces, and further, a space from which there is no clear exit strategy. As anthropologist Ilana Feldman has noted in reference to previous humanitarian spaces in Palestine:

A key challenge of the humanitarian condition is that it goes on and on. Available services can never be adequate to meet people's needs. And no matter how much effort goes into reforming service delivery, humanitarian systems are always partly defined by failure, the causes of which often lie outside the humanitarian domain. In the Palestinian case, the necessity of humanitarian services over a person's full life cycle confirms the failure of political actors to address the core problems [75].

COGAT's designation of the Zone as the ‘only safe area’ in Gaza allowed the IDF to demand the forced displacement of the Palestinian population of Gaza into the Zone under the guise of facilitating aid [27]. Despite this designation, and the widespread understanding of the Zone as a safe haven, it has been deliberately targeted multiple times, most notably during an Israeli operation to rescue six Israeli hostages, which resulted in the deaths of 273 Palestinians [85].

Humanitarian aid groups have inadvertently legitimized the Humanitarian Zone by operating within its confines largely without questioning its existence and constructing medical field hospitals (MFHs) that provide immediate relief but have simultaneously paved the way for new forms of Israeli control [84]. These MFHs, typically operational for less than 6 months, deliver lower‐quality care compared to the standard within Palestinian hospitals and rely on international aid workers, who often serve short rotations of 2 months. This instability and inconsistency in services has further disrupted the healthcare system, with Palestinian workers being drawn from areas outside the Humanitarian Zone where they were needed most, leaving those unwilling to abandon their homes with diminished healthcare options. These actions have enabled Israel to sever northern Gaza from the south [86], effectively fragmenting Palestinian society within the Strip. This division has been particularly devastating given that the ability of a community to remain on their land depends on access to functioning health infrastructure [65].

By concentrating their operations within Gaza's designated Humanitarian Zone, a decision that is framed as a protective measure for international staff, aid organisations have tacitly accepted that the lives of foreign workers are worth more than those of Palestinians [33]. As anthropologist Didier Fassin argues, humanitarian practice often operates within a framework of *ontological inequality*—a moral hierarchy of whose lives may be risked. In Gaza, this hierarchy is in sharp relief: international humanitarian aid workers are relatively protected and Palestinian lives are lives that can be sacrificed [87]. This disparity is evident in the differing institutional responses to harm: when international aid workers, who voluntarily enter Gaza, face injury or death, their suffering prompts immediate international concern and policy reassessment [88]. In contrast, Palestinian aid workers, who have no choice but to remain and endure constant danger, receive little acknowledgment when harmed or killed [89]. One of the most notable examples in Gaza is the deaths of three international workers belonging to the World Central Kitchen (WCK), which led to widespread media coverage and worldwide condemnation [90], including by then‐US President Joe Biden [91]. The deaths of these international workers by an Israeli airstrike, while inside a marked humanitarian car, served as unquestionable evidence of indiscriminate, unprovoked Israeli fire. Condemnations were issued largely without reference to the mounting death toll of Palestinian humanitarian aid workers, with the Gaza Strip recording the highest number of aid worker fatalities in a single crisis ever recorded between 7 October 2023 and 23 September 2024 [92]. On 7 August 2024, another WCK staffer, a Palestinian warehouse worker in central Gaza, was killed in an air strike. His death received scant media coverage [93].

Many humanitarian NGOs have participated in documenting and reporting the atrocities occurring in Gaza, often taking on the duty of ‘bearing witness’ [87] There is obvious value in this work, but the discursive space it takes often precludes Palestinians' narration of their own lived experiences [94]. Accordingly, Palestinians are represented—even, and sometimes especially—by humanitarian groups, in reductive and caricatured terms, either as entirely helpless victims in need of humanitarian aid, or (mostly in the case of men and boys of fighting age) as likely ‘terrorists’ unworthy of protection [95]. Western aid workers are generally presented as the sole credible voices, frequently appearing in Western media outlets to share ‘what they saw’ [96], privileging their supposedly objective, Western, universal perspective over the lived realities of Gazans. Part of this presumed ‘objectivity’ is a result of the presumed and revered ‘neutrality’ of their organisations. Figures relating to the number or percentage of women and children killed in Gaza function as the main tools to advocate for a ceasefire [97], manufacturing an image of innocence in Gaza, that is, of a woman or a child who is assumed, by definition, to not be a Hamas operative. The image of Palestinian women and children as innocent victims is often used to prioritize their access to humanitarian aid, while Palestinian men are racialized as potential threats. This narrative, widely embraced by many Western aid workers and news outlets, renders the indiscriminate killing and injuring of Palestinian men in Gaza largely invisible and therefore unchallenged [98].

The lack of strong opposition to Israel's occupation, blockade, and genocide in Gaza has permitted Israel to humanitarianise its oppression, increasing the scale and depth of Palestinian suffering [99]. International aid groups focus on putting pressure on Israel to permit the opening of new international field hospitals, ignoring the need for efforts to rebuild Palestinian hospitals and medical schools. This approach only serves to further entrench the humanitarian framing of the Israeli occupation of Gaza. A new fragmented healthcare system in post war Gaza will heavily rely on international aid, leaving Palestinians at the mercy of aid groups and the political contingencies that determine the scope of their work. Critical care, like advanced surgeries or cancer treatment, may soon cease to exist entirely in the Gaza Strip [100], and the many Palestinians disabled due to the loss of limbs or other serious physical injuries may be left without proper long‐term care and rehabilitation [101]. By the end of 2023, cancer patients were already left to suffer without treatment, as power outages, scarcity of medicines, and the need to triage those injured in strikes diverted limited resources away from long‐term care. Salem Khreis, a 40‐year‐old leukemia patient in Gaza shared his experience with *Al Jazeera*: ‘There's no medicine or treatment … I can't explain how terrible the pain is’ [82].

Israel's deliberate production and strategic manipulation of humanitarian crisis in the oPt has been met, for the most part, with silence by the international community, broken only by the routine dispatch of additional Western humanitarian aid into Palestinian communities [102]. This tacit endorsement of Israel's control over the humanitarian narrative reveals the neo‐colonial logic underpinning aid in Palestine, where humanitarian mechanisms increasingly serve to stabilise and legitimise Israel's occupation of Gaza. Since 7 October 2023, this dynamic has only intensified [68].

The acceleration of the humanitarianisation of Gaza's healthcare system represents a profound bioethical challenge. By reconfiguring healthcare delivery through a humanitarian lens rather than as a sustainable system of rights‐based care, scarcity is institutionalized as the operational norm. Scarcity is core to clinical ethics, and it generates cascading bioethical dilemmas: extreme triage decisions that force clinicians to violate core medical and personal principles; the undermining of patient autonomy when care options are severely constrained; the disruption of provider‐patient relationships when humanitarian actors rotate frequently; and the politicisationed of health through its dependence on aid mechanisms. One Gaza doctor at Al‐Shifa Hospital described having to let a patient die due to resource constraints: ‘I had to stop the resuscitation of a patient who went into cardiac arrest in the dialysis unit, because if she made it back to life, we had no ventilator to offer her [103]’.

Most troublingly, this framework normalizes inadequate care as acceptable under ‘crisis conditions,’ even as these conditions persist indefinitely, suspended in a state of managed emergency. Global political actors, meanwhile, refrain from meaningful intervention, deferring instead to humanitarian organisations who are presumed to be able to avert total collapse. This deferral not only displaces accountability but entrenches a system in which ethical burdens fall disproportionately on local healthcare workers, forced to navigate impossible moral choices within artificially constrained parameters. Crucially, the structural forces producing this scarcity—occupation, blockade and geopolitical abandonment—remain largely unexamined within mainstream bioethical discourse.

The implications of this humanitarianised healthcare regime can be better understood by situating the realities just described within a theoretical framework that interrogates how power operates through the calculated management of life and death, and how Gaza's medical infrastructure has become a site where sovereignty is exercised through the regulation of suffering. In the next section we take on this task.

## 3 Applying ‘Necropolitics’ in Gaza

In 1979, Edward Said wrote that understanding Palestine means confronting the fact that the situation is characterised by a ‘contest between an affirmation and denial [104].’ Palestinian life is viewed by the Israeli government and its Western allies as a reality that must be refused, an inconvenience in need of careful and continual management. That management has taken many forms, running the moral spectrum from surveillance, containment, and harassment all the way through to genocide [105]. At times, Palestinians have been permitted to live provided they consent to do so under conditions that severely restrict their quality of life; at other times, Palestinians have been summarily killed in large numbers. The second situation is the context from which we write, but the first situation has been the more everyday experience of most Palestinians over the last half century. In this section of the paper, we introduce the idea of necropolitics as a way of understanding the business‐as‐usual oppression of Palestinians. We then show that while necropolitics may appear to be an inadequate lens for understanding genocide, the destruction of the health system of Gaza, as outlined in section 1, can be viewed as a critical step in ensuring an enduring necropolitical reality.

### 3.1 Introducing Biopolitics and Necropolitics

‘Biopolitical’ theories relate to the intersection of politics and human biology, emphasizing how states and other powerful entities regulate and control populations by making interventions in relation to health, reproduction, and bodily autonomy. Bodies are the units of populations: if their biology can be managed, populations themselves can be regulated. Typically, biopolitical control involves limiting or optimising the physical and mental health, economic productivity, and fertility of ‘undesirable’ and ‘desirable’ populations. Biopolitics therefore offers important insights as to the ways in which racialised people subject to the violence of colonial states are ‘managed’, and it is an especially useful theoretical frame in cases where interventions into the health system are well‐documented.

Achille Mbembe's theory of necropolitics was developed as a response to, and an expansion of, among other theories, Michel Foucault's concept of biopolitics, Giorgio Agamben's notion of the ‘bare life’, and Judith Butler's work on grievability. In what follows, we briefly rehearse the most relevant aspects of these theories, describe the central features of Mbembe's theory, and justify this choice of theoretical framing.

Foucault's biopolitics describes the way in which states exert control over *life*, managing populations through institutions and regulatory mechanisms which regulate individual bodies [2]. Mbembe built on Foucault's conception of biopolitics by pointing out that power over bodies is also exercised through the control of *death*, especially in contexts of colonial and postcolonial violence [106]. Mbembe also builds on Agamben's concept of ‘bare life’, a concept which refers to a life stripped of political rights, in which a person exists solely as a biological being, often under the control of a sovereign power [6]. Mbembe extends this idea to examine how modern states exert control through the systematic exposure of certain populations to death and violence, intentionally producing conditions of ‘bare life’ for whole groups. Necropolitics is also closely related to Judith Butler's work on the politics of disposability and exclusion. Butler's notion of ‘grievability’ captures the way in which certain lives are deemed worthy of mourning while others are not. They argue that in times of war, the enemy's dead are often depicted as ungrievable, meaning their lives are not recognized as valuable or worthy of mourning [107]. This notion of grievability is central to understanding the differential value of lives. As Butler writes: ‘Without grievability, there is no life, or, rather, there is something living that is other than life [108].’

In his development of the theory of necropolitics, Mbembe synthesises and builds on these ideas to explore how sovereignty is enacted through the power to decide who may live and who must die, particularly in contexts of racialized and colonial violence. One of the central insights of the theory is in the recognition that people enslaved in the racist regimes of European colonialism were less often killed than ‘kept alive but in a state of injury, in a phantom like world of horrors and intense cruelty and profanity [106]’. Killing is not always easy, tolerable within one's broader moral community, or expedient; sometimes something close to death is a preferable form of control. In other words, in some contexts oppression proceeds not primarily through outright murder, but instead by producing ‘death‐worlds, new and unique forms of social existence in which vast populations are subjected to conditions of life conferring upon them the status of living dead [106].’ Necropolitics is the logic and necropower is the mechanism through which ‘death‐worlds’ are secured. It is the strategic use of political, economic, and military force to determine who may live and who must die, governing populations through exposure to death and the withholding of care. It captures profound and intentional forms of deprivation that are often (understandably) sidelined by counts of lost lives and limbs: A 21‐year‐old Gazan woman described the impact of an airstrike in 2024 on her plans to become a teacher: ‘That day I lost more than just my leg. My dreams vanished [109]’

One common application of necropolitics is to the lives of necessitous migrants in the UK and other Global North states, who live severely constrained lives of intentional, open brutality at the hands of hostile immigration regimes, which has been referred to as ‘conspicuous marginalization [110, 111]’. Applying necropolitics in the case of Palestine is not novel; Mbembe has always taken it to be a paradigm case, writing that the ‘most accomplished form of necropower is the contemporary colonial occupation of Palestine [106]’￼

### 3.2 Necropolitics in Times of Genocide

Necropolitics accurately describes the quotidian oppression experienced by Palestinians living under Israeli occupation in the Gaza Strip. Consider the fact that Israeli defence ministry files released under court order in 2012 showed that the food calories permitted to enter Gaza between 2007 and 2010 were precisely calculated by the Israeli government to provide just enough energy per person to prevent malnutrition, and no more [112]. Palestinians in Gaza were allocated enough food for physical survival (assuming perfect conditions of distribution and preparation) but not to ensure their thriving or to provide choice and security.

Necropolitics seems, at first sight, less apt as a way of theorising a genocide in which at least 62,614 Palestinians have been killed and a further 14,222 are missing, almost all of whom must be presumed dead [113]. Recall that Mbembe describes as necropolitical those ‘new and unique forms of social existence in which vast populations are subjected to living conditions that confer upon them the status of the living dead [106].’ Can necropolitics–a theory of social and political death–shed light on a situation which consists of so much *actual* death? And what should we make of Israel changing course from its attritional strategy of ‘slow violence’ to such immediately catastrophic ‘fast violence [114]’?

Making sense of this shift in the intensity and pace of violence requires an understanding of why necropower, which suppresses life until it becomes close to, but not quite, death, is deployed in the first place. There are several reasons to note. First, there is a general favouring in almost every moral system of life over death, regardless of the quality of the life under consideration. (This moral intuition grounds much of the automatic, pre‐theoretic opposition to (assisted) suicide and abortion.) Anthropologist Didier Fassin refers to this bias as ‘biolegitimacy’, defined as ‘the recognition of life as a supreme good in the name of which any action can ultimately be justified [4].’ Second, and relatedly, necropolitics is often favoured precisely because it is not as morally objectionable as genocide, which is not (so easily) forgiven by the broader moral community. Third, producing necropolitical conditions means leaving most of the population not only still alive but also so desperate as to be easily exploitable. Israel has long seen Palestine as an important economic resource, and the containment of the Palestinian economy inside the Israeli economy, on terms of subjugation, has been used as a further tool of dispossession, and to routinely execute collective punishment [115]. As Moshe Dayan, Israel's defence minister during the 1967 6‐Day war, said, the oPt were intended to be ‘a supplementary market for Israeli goods and services on the one hand, and a source of factors of production, especially unskilled labor, for the Israeli economy on the other' [116].

Israel's necropolitical reality in Gaza became a genocidal one because, on 7 October 2023, Palestinians staged an act of violence so harmful (relative to previous acts of Palestinian resistance) and so unexpected that Israel no longer felt bound by the necropolitical terms of engagement it had previously observed. Specifically, the restraint once exercised to avoid crossing a moral line that might lead to ostracism was now deemed unnecessary. Among Israel's allies, all Palestinian resistance amounts to ‘terrorism’, a moral wrong taken to be so heinous as to merit unrestrained punishment and disproportionate measures that can be described as ‘self defense’. On 9 October, Israeli Defence Minister Yoav Gallant announced that: ‘there will be no electricity, no food, no water, no fuel, everything will be closed. We are fighting against human animals, and we are acting accordingly.’ And when UK Prime Minister Keir Starmer was asked, later that month, whether cutting off Gaza's electricity and water supply was a proportionate response to the events of 7 October, he responded: ‘I think that Israel does have that right, it is an ongoing situation, obviously everything should be done within international law but I don't want to step away from the core principles that Israel has the right to defend herself [sic] [117].’ Mass killing therefore became morally excusable because it could be convincingly portrayed as a response to an unprovoked massacre, rather than the intensification of a regime of violence.

Importantly, the necropolitical regime did not end when the genocide began. Rather, the two now coexist. According to the logic of biolegitimacy, necropower is not as morally objectionable as direct military might, which means that forms of destruction which will guarantee the production of long‐term ‘death‐worlds’ are not judged as harshly or straightforwardly as simply killing. Genocide can therefore enable the operation of necropower by operating as a sort of moral decoy. Accordingly, Israel's necropolitical regime in Gaza has intensified along with the genocide, and nowhere is this more obvious that in the deliberate mass destruction and humanitarianisation of the health system, as discussed in Section 2, as well as the destruction or control of the determinants of health: food, water, fuel, shelter and sanitation [9]. The consequences of this dismantling of the fabric of wellbeing in Gaza will have long term effects. Scientists estimate that 186,000 people could die as a result of malnutrition, poor sanitation, and the inability to access healthcare [118]. The destruction of healthcare and its determinants since 7 October should be seen as a process of reconfiguring Gaza as a new humanitarian, and therefore necropolitical, space.

In a basic sense, genocide is always necropolitical in that not everyone is killed, and those who survive must either become refugees and face necropower elsewhere, or else remain and inhabit severely diminished ‘death‐worlds’ that are quite literally filled with death, in the form of cadavers, funerals, and grief, but also the symbolic death of communities (through the destruction of places of learning, leisure, and worship), and the ruins of the physical environment: the shells of buildings, and the loss of farmlands, including ancient olive groves [119].

Necropolitical conditions are not sufficient for genocide, but they are a necessary precursor. This claim can be understood in several ways. When the health of a population has been systematically undermined over time, the devaluation of that population becomes normalised, and the (lack of) response from national and international actors is already rehearsed. Moreover, the physiological vulnerability of those subjected to prolonged deprivation renders them more susceptible to death when further violence is introduced. The deprivation of health and the deprivation of life exist on a continuum. Necropolitics is a condition for genocide and is evidenced in its details, such as the broad acceptance of an extremely uneven death toll and prisoner‐hostage exchange ratio.

It is in this context that we return to the question that opened this subsection: why deploy the theoretical framework of necropolitics in relation to the genocide in Gaza? Necropolitics helps us understand Israel's assault on health in Gaza in a way that resists framing the genocide and the wholesale destruction of healthcare infrastructure as a rupture. Rather, the systematic undermining of health has long served to position Palestinians in proximity to ‘death worlds’: zones of structural abandonment, severe deprivation, and extreme, continual violence that render life precarious and disposable.

While this paper treats the necropolitical reality in Gaza as distinct from the current genocidal phase, we recognise that long‐term regimes of control, deprivation, and subjugation, especially those that target the conditions of life, are themselves genocidal, relative to any historically and legally grounded definition. The UN Convention defines genocide as acts committed with intent to destroy, in whole or in part, a national, ethnic, racial or religious group, including not only killing but also inflicting conditions calculated to cause ‘serious bodily or mental harm to members of the group [120]’. Necropolitics, understood as the governance of death and the calculated exposure of populations to slow violence, is therefore a key modality of genocide.

The humanitarianisation of healthcare in Gaza represents a strategic extension of necropolitical governance, wherein the meeting of basic needs is no longer understood as the realisation of fundamental rights but as an act of charity, and political claims to sovereignty, self‐determination, and accountability are replaced with a distracting and depoliticised framework of crisis management and emergency response. Humanitarian healthcare provision is thereby complicit in sustaining the very necropolitical conditions it sets out to alleviate, as it normalizes unending emergency while failing to address the violence that necessitates intervention in the first place.

Moreover, humanitarianisation serves as a transitional mechanism between necropolitical conditions and genocide. By reducing an occupied population to biological life requiring external sustenance for survival, humanitarianisation limits Palestinians' political agency and renders them perpetual recipients of aid rather than rights‐bearing subjects. This reduction to bare life—where existence is measured in caloric intake and the minimal health access required to continue living—helps to create the conditions where mass death becomes morally tolerable to international observers. The humanitarian lens thus obscures the political dimensions of suffering, presenting genocide as an unfortunate humanitarian crisis requiring technical solutions.

## 4 Towards a Biopolitical Bioethics

Bioethics and biopolitics are generally taken to be distinct approaches to understanding the complex nexus of biological life, power, and justice. Bioethics tends to focus on the moral dimensions of medical and biological interventions, focusing on individual rights, informed consent, and ethical decision‐making within healthcare and scientific research contexts. By contrast, biopolitics studies how political power systematically manages, regulates, and governs human populations through biological mechanisms. Where bioethics tends to focus on individual moral choices, biopolitics interrogates the broader structural mechanisms through which power monitors and constrains biological existence.

Like many disciplinary divisions, the boundary between bioethics and biopolitics is based not on a clear methodological or content‐based difference, but rather on arbitrary genealogical features. Bioethics arose from the need to regulate medical practice and research; biopolitics from political and historical studies of governance, sovereignty, and the operation of power. Bioethics was developed largely in Anglo‐American settings, with the tools of analytic philosophy; biopolitics has been strongly influenced by French and Italian philosophers working in the continental tradition.

There has been a concerted effort in recent years to engage in more politically informed bioethical inquiry and‐or to demand that the discipline broaden its scope beyond the contingent and sometimes arbitrary bounds imposed by tradition [4, 7, 8, 9, 10, 11, 12, 13, 14, 15, 121]. We proceed in this vein, noting that the strict delineation between bioethics and biopolitics becomes untenable in contexts of extreme violence and structural oppression, where attempting to maintain disciplinary boundaries can impede meaningful ethical analysis. Israel's genocide in Gaza is one such situation of grave moral urgency, and (at least) two important questions arise for bioethicists:

1. What is the role of bioethics and bioethicists in relation to situations in which the life and health of an entire social group are threatened?
2. At what point does bioethics become biopolitics? Should bioethicists ‘stay in lane’?

Our responses to these questions are related: bioethics must be strongly informed by biopolitics, or else we are severing situations of moral concern from the contexts that produce them, and cannot hope to understand them, answer them properly, or recognise similar situations in order to prevent them.

In a 2025 editorial in *Bieothics*, Udo Schuklenk writes that bioethicists should focus on ‘specific bioethics issues’ as they relate to larger political events—for example, ‘the professional obligations of healthcare professionals to enemy combatants, terrorists with and without inverted commas, military attacks on healthcare facilities suspected of being used as shields by a group in the conflict’—but avoid weighing in with broader political analysis, since, for example, ‘who the guilty party is in terms of what caused the Gaza conflict is not a bioethics issue [122].’ (He is not the first to make this sort of argument. Writing in 2006, Jeffrey Kahn worried that ‘Once we take a position and begin to advocate on behalf of a specific issue, we risk becoming partisans [123]’, a statement which is presumably only worrying if the position is not arrived at via robust moral arguments. We are all, presumably, partisans to the cause of taking positions that are morally justifiable.) Bio*political* analysis, then, is presumably off limits. Shuklenk's claim seems to be that bioethicists' expertise is limited to these specific clinical dilemmas and they should work within this expertise. But bioethicists' expertise is normative analysis applied to health and the body, which can be interpreted very broadly, and presumably should be, in the absence of strong reasons for a narrow interpretation. Every time a new technology with some biological or medical application is developed, no matter how complex, bioethicists quickly develop entirely new expertise with regard to that technology, and then apply their skills in normative analysis to their new area of expertise. Bioethicists are presumably just as capable of acquiring new expertise in relation to an unfolding geopolitical issue with serious health ramifications, or if they aren't, this shortcoming should be addressed within bioethics teaching and training.

Our perspective is closer to that of Lederman and Lederman, who, in a paper urging bioethicists to recognise their responsibilities towards those facing dire public health crises in Yemen, define bioethics much more broadly than Schuklenk, writing that ‘social justice that has any bearings on human health (which is plausibly always the case) is the business of bioethics [7]’ Further, they argue that writing about situations of vast human suffering are not only the purview of bioethics, contra Schuklenk, but ought to be the discipline's priority, because: ‘All we write about in bioethics is urgent and important, but some topics are more urgent and important than others [7].’ Similarly, as Shahvisi writes in a 2024 editorial for the *Journal of Medical Ethics*, the sort of contained bioethical questions that the likes of Schuklenk take to be the only appropriate work for bioethicists are not really contained at all; their containment is enacted by bioethicists in order to make their work easier, and can easily lead to bioethicists missing the point entirely. It is well and good, Shahvisi writes, to think of bioethics as dealing only with ‘problems clinicians must grapple with within the walls of the hospital’, but ‘What happens when the walls of the hospital are blown away? […] What should medical ethicists do when extreme scarcity arises because the entire health infrastructure of a place is being deliberately destroyed? [9]’ This, we contend, is the point at which bioethicists need to draw on biopolitical analysis in order to understand why the bioethical issues arise in the first place.

A final point of note is that Palestinian scholars and activists have employed biopolitical, and specifically, necropolitical, frameworks to interpret and articulate their lived experiences under occupation, providing crucial insights into power dynamics that determine who may live and who may die [114]. Given that those most affected by these conditions have found analytical value in understanding their situation through this theoretical lens, the application of necropolitical analysis deserves serious consideration by bioethicists engaging with questions of health and life in Gaza.

In the present study, a history of the humanitarianisation of health provision in Gaza and the necropolitical conditions under which people in Gaza have lived for decades are critical context to any bioethical study of, say, the moral dilemmas clinicians face as they navigate the task of providing healthcare to those who remain in Gaza. The ethical challenges for healthcare workers in Gaza are inseparable from the biopolitical conditions under which they operate. Clinicians are routinely forced to make decisions that violate standard ethical norms, because the infrastructure required to uphold those norms has been systematically dismantled. Triage, which is usually an emergency protocol, becomes a daily necessity; informed consent is compromised by lack of options; and the broader duty to deploy one's skills to provide essential care is undermined by the impossibility of meeting even basic medical needs or of guaranteeing one's own personal safety or that of one's colleagues. The reliance on international aid further complicates this picture. While essential for survival, humanitarian aid is shaped by donor priorities and political constraints that undermine Palestinian autonomy and fragment local health governance. Healthcare workers therefore grapple with the more obvious clinical dilemmas while navigating a humanitarian system that is necessarily complicit with the occupying power. These challenges demand a bioethics informed by biopolitics.

We therefore encourage readers to see the analysis in this paper as a work of bioethics, and we encourage other bioethicists to make similar use of biopolitics in their work in this area and in comparably urgent geopolitical crises.

## 5 Practical Recommendations for Resisting Humanitarianisation and Necropolitics in Bioethics and Beyond

In this final section, we list some practical measures that medical practitioners and NGO health workers can take to resist humanitarianisation and necropower. We hope that bioethicists will see these recommendations as topics of ethical interest which might be fruitfully subject to biopolitical bioethics analysis in order to encourage the field to think about the inherent role of politics in the ethics of healthcare delivery.

First, those working within and studying healthcare should make efforts to document and publicly announce instances where medical resource scarcity is political rather than inevitable, distinguishing between true resource constraints and manufactured ones. This documentation should record not only what care was provided but what care would have been indicated under standard medical protocols. Such counter‐narratives challenge the normalisation of inadequate care standards in politically complex environments. Relatedly, medical practitioners should engage in strategic documentation of healthcare obstruction that meets evidentiary standards for future accountability processes. By meticulously recording instances where political decisions directly impact healthcare delivery—such as delays at checkpoints, denial of medical transfers, destruction of buildings and equipment, or restrictions on medical imports—practitioners create an archive that challenges impunity and the normalization of healthcare deprivation as a political tool.

While many organisations such as Médecins Sans Frontières already collect statistics on attacks on healthcare and access barriers, these efforts are often constrained by humanitarian mandates, donor pressures, and security risks. Documentation sometimes remains depoliticised or disconnected from solidarity‐based frameworks of resistance. We therefore recommend that such documentation be explicitly designed to support Palestinian‐led advocacy, linked to legal and political accountability mechanisms, and integrated into broader strategies for resisting humanitarianisation and necropolitics. This requires a shift towards co‐produced, action‐oriented, and politically engaged indicators. Documenting evidence of atrocities must not result in the ‘humanitarian reduction of the victim [87]’. Rather, they should be linked to a reflexive and action‐orientated solidarity [124]. Second, medical practitioners and NGO health workers might consider their responsibility to take on the personal or organisational risk of delivering more effective medical aid outside of Israel's restrictions. This could involve collaborating with local health workers to navigate restrictive barriers, such as coordinating the covert delivery of critical medical supplies or establishing decentralised care networks that operate independently of colonial oversight. For instance, the Palestinian Medical Relief Society provides essential healthcare services through mobile clinics and community health centres that often work outside the constraints imposed by Israeli restrictions [125]. These mobile units are crucial in delivering medical aid to underserved areas, and those areas such as Northern Gaza that are the focus of the Israeli military's genocidal and ethnic cleansing efforts. Effective international NGO collaboration means supporting, rather than supplanting, the leadership of local Palestinian health professionals, including through risk‐sharing agreements, financial and logistical support (e.g., for mobile care units), and international advocacy to protect these activities under international law.

Healthcare workers might also establish networks of solidarity with their Palestinian counterparts that bypass official humanitarian channels when these channels reinforce dependency. These horizontal relationships between medical professionals enable knowledge exchange, resource sharing, and mutual support that operates outside the vertical donor‐recipient hierarchies typical of humanitarian interventions. These networks also facilitate the preservation of Palestinians medical knowledge and locally appropriate healthcare approaches that humanitarian standardization often displaces. Relatedly, practitioners should prioritize building sustainable health infrastructure even within emergency response frameworks. This includes investing in local healthcare worker training, developing context‐appropriate medical technologies, and designing interventions with clear exit strategies that transfer capabilities to local systems. By refusing the perpetual emergency framing, healthcare workers insist on the right of all communities to stable, developing healthcare systems rather than indefinite humanitarian life support. The Gaza Educate Medics Initiative [126], launched by PalMed Academy, aims to rebuild medical education in Gaza by establishing a virtual medical college. This programme, supported by academics and consultants worldwide, allows 2500 medical students in Gaza to continue their education and graduate from their respective institutions while preserving accreditation.

Finally, healthcare workers should reframe their ethical responsibilities to include challenging the structural violence that produces preventable suffering. This expanded conception of bioethics moves beyond treating the symptoms of political crises to addressing their causes, recognizing that attempts at ‘neutrality’ in contexts of severe power asymmetry often serves to perpetuate rather than ameliorate suffering. By explicitly acknowledging the political determinants of health alongside social ones, practitioners reclaim healthcare as a site of resistance to necropolitical logics rather than serving as unwitting accomplice to them. Part of this is to recognise that the necropolitical logic of Israeli military actions have not only inflicted physical destruction but have also sought to sever the profound connections Palestinians have with their land and each other, exacerbating trauma and undermining communal well‐being. The restoration of health requires that public health policy, and healthcare practitioners, actively resist Israel's occupation of Palestinian land. Partnerships with organisations such as the Palestinian Agricultural Relief Committee (PARC) would allow for the integration of liberatory psychosocial support within agricultural projects. Healthcare practitioners also have a role to play in projects that restore the land and culture, such as those bringing back olive groves destroyed by the Israeli military. PARC's previous power‐building social interventions with small‐scale producers in post‐catastrophe Gaza [127], in collaboration with NGOs, offers a useful model for future psychosocial work.

These approaches invite bioethical analysis that goes beyond traditional clinical ethics to examine how political frameworks shape the very conditions under which ethical decisions must be made. By bringing biopolitical analysis into bioethics, the field can develop more nuanced frameworks that account for how power relations determine which populations receive care, under what conditions, and toward what ends. This expanded bioethical lens would recognize that in humanitarianised contexts, the most profound ethical challenges often precede and shape clinical decision‐making rather than emerging solely within it. Ultimately, bioethicists’ main role in the Palestinian‐Israeli conflict must be advocating for the end of the Israeli occupation of Palestine, and most urgently the end of the siege on Gaza, which is destroying the health of an entire population.

## 6 Conclusion

In this paper, we have reviewed Israel's destruction and humanitarianisation of the health system in Gaza and have analysed this regime of control and subjugation through the lens of necropolitics. We have argued that Israel's extended policy of restricting Palestinian access to care, destroying healthcare infrastructure and rendering healthcare infrastructure dependent on external, conditional aid is not merely an unfortunate by‐product of war or occupation, but a deliberate strategy for producing ‘death‐worlds.’ We have questioned the uncritical application of humanitarianism and have criticised the failure of bioethics to respond adequately to the manufactured health catastrophe now unfolding in Gaza, and have urged the politicisation of humanitarian and bioethical efforts, arguing that it is necessary for scholars, NGO health workers, and medical practitioners to embrace a biopolitical understanding of bioethics. We have offered some suggestions as to how these actors can resist humanitarianisation and the operation of necropower in Gaza.

This paper was written against the backdrop of the genocide in Gaza. It is difficult to calculate or comprehend the degree of destruction in Gaza, what its consequences will be for the lives and health of Palestinians, and what ‘ethics’ will look like in its aftermath. Our work is an attempt to understand and memorialise a small part of the ongoing nakba [128].

## Conflicts of Interest

The authors declare no conflicts of interest.

## Data Availability

The data that support the findings of this study are available from the corresponding author upon reasonable request.

## Endnotes

1 The Testimony can be found here: https://www.aljazeera.com/news/2023/10/12/war-crime-gaza-medics-say-israel-targeting-ambulances-health-facilities. Many other similar cases unfortunately occurred like the Rafah paramedic massacre, which resulted in the deaths of at least 15 Palestinian aid workers.

2 In February 2025, US President Donald Trump further pushed the idea of mass expulsion. President Trump expressed a vision to redevelop the Gaza Strip as a ‘Riviera of the Middle East’, and the forced expulsion of 2 million Palestinians from Gaza.
